# SOD-FE: a supervised outlier detection and feature engineering approach for student dropout prediction

**DOI:** 10.1038/s41598-026-56591-6

**Published:** 2026-06-09

**Authors:** Eissa Alzabidi, Önder Yakut, Sabah Saad, Oğuz Fındık

**Affiliations:** 1 Department of Computer Engineering, Hodeidah University, Hodeidah, Yemen; 2 Department of Computer Engineering, Karabük University, Karabük, Turkey; 3 Department of Information System Engineering, Kocaeli University, Kocaeli, Turkey

**Keywords:** Student dropout prediction, Supervised outlier detection, Feature engineering, Machine learning, XAI, Applied mathematics, Computational science, Computer science, Information technology, Statistics

## Abstract

Student dropout in higher education creates academic and socio-economic challenges for institutions and students. Effective early prediction models are essential to identify at-risk students and implement timely interventions. This paper proposes SOD-FE, a supervised machine learning approach that combines label-aware outlier detection with feature engineering to enhance dropout prediction. The approach integrates interquartile range (IQR) based outlier detection with mutual information and Pearson correlation to identify and mitigate the impact of outliers before constructing the final model. Then, a feature selection strategy is applied to refine the dataset. The approach is evaluated through experiments on two real-world datasets (Portugal and Slovakia) utilizing five classification algorithms, including Random Forest (RF) and Extreme Gradient Boosting (XGB). Performance improved markedly with the RF classifier, achieving F1 scores of 98.09% and 98.33% on the Portugal and Slovakia benchmark datasets, respectively, under 5-fold cross-validation. Furthermore, the SOD-FE approach outperformed the baseline RF models by 7.46% and 3.37% in F1 score across the two datasets, demonstrating highly competitive results that align with or exceed the highest reported performances in recent literature by up to 7.33%. The proposed approach also incorporates explainable AI techniques (SHAP) to enhance model transparency and support data-driven educational policy. These findings show the significant potential of the SOD-FE method for improving student retention and early intervention systems in educational institutions.

## Introduction

Student dropout in higher education is a serious problem that affects not just the students but also educational institutions^1,2^. Recent studies show that high dropout rates lead to financial and reputational losses for institutions through increased operational costs for institutions, lower graduation rates, reduced enrollment income, and underutilized public investment^3–5^. At the individual level, dropout is associated with lower employment prospects, lifetime earnings and health^6–9^. These quantified impacts underscore the urgency of developing accurate and scalable early warning systems capable of identifying at-risk students in a timely manner^4,10–12^.

In recent years, the application of machine learning (ML) techniques in the education sector has garnered significant attention, particularly in the context of predicting student academic success^13^.The application of these techniques can be used to aid in the development of early warning systems and identify at-risk students using demographic, socioeconomic, macroeconomic and academic datasets that have been developed^14^. Several ML methods were used in previous studies to predict student performance, such as decision tree (DT), logistic regression (LR), SVM, and ensemble methods such as RF, XGB, etc. In addition, various resampling techniques such as SMOTE^15^, ADASYN^16^, etc., were utilized. These algorithms proved to be effective in the binary classification of students as graduates or dropouts^17,18^.

This paper presents a proposed supervised outlier detection and feature engineering (SOD-FE) approach developed to improve the effectiveness of student dropout prediction models. The primary objective of this approach is to enhance data quality and model performance by applying target-aware outlier filtering and informed feature selection prior to training the final classifier. Unlike traditional unsupervised outlier detection techniques, the proposed method utilizes label information in conjunction with mutual information and Pearson correlation to identify the most relevant features and eliminate instances that are likely to introduce noise or bias^19^. Outliers are detected using an Interquartile Range (IQR)-based statistical filter, which is tuned concerning class distribution, ensuring that critical patterns, particularly those related to minority dropout classes, are preserved^20^.

In addition to its foundational elements, the SOD-FE approach offers several key contributions. First, it introduces a supervised outlier score as an additional predictive feature, allowing models to benefit from label-informed anomaly detection. Second, a parametric optimization strategy is adopted to fine-tune the filtering process based on both class relevance and feature informativeness. Third, it employs a dual-stage feature selection mechanism, combining Random Forest Importance (RFI) and Recursive Feature Elimination (RFE) to improve interpretability and reduce redundancy. The approach further incorporates explainable AI techniques (SHAP) to highlight influential features and support transparent, data-driven decisions^21^. Moreover, by integrating resampling methods such as SMOTE and ADASYN, SOD-FE enhances model robustness against class imbalance, achieving highly competitive F1 scores exceeding 98% on the evaluated datasets (Portugal and Slovakia), which ranks among the highest reported performances in the current literature.

**Fig. 1:**
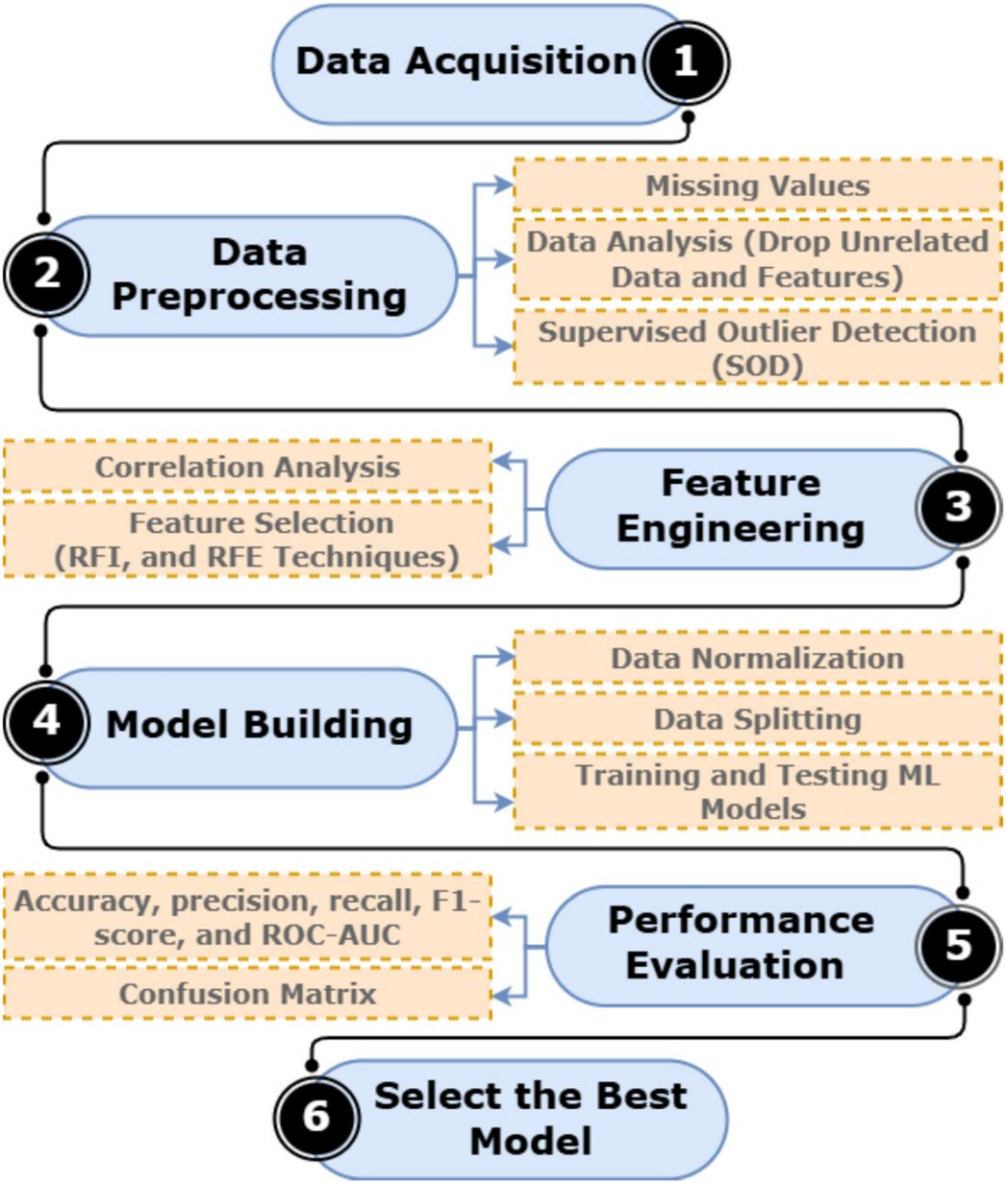
The proposed approach (SOD-FE).

These contributions are compatible with the main stages of the predictive modeling process for student academic success, as shown in Fig. 1. The key stages include data acquisition, data preprocessing, feature engineering, model building, model evaluation and best model selection. Data acquisition involves gathering relevant datasets from institutional sources. The acquired data then undergoes data preprocessing, which includes handling missing values, encoding categorical variables and using the proposed Supervised Outlier Detection (SOD) method. Following this, we move on to the feature engineering stage, which focuses on enhancing the dataset and improving the model’s predictive capabilities by selecting, transforming, and creating new features that enhance the models predictive^22^. Next, model building involves training several classification models, including Logistic Regression (LR), Support Vector Machine (SVM), Random Forest (RF), Extreme Gradient Boosting (XGB), and Multi-layer Perceptron (MLP). The performance of these models is assessed through the supplied test set, and cross-validation, using hold-out and 5-fold cross-validation settings, was performed. Finally, the results are analyzed in detail, with performance metrics visualized to facilitate interpretation.

## Related work

Recent advances in ML have significantly improved the ability to predict student dropout by leveraging diverse datasets, algorithms, and evaluation methodologies. Traditional ML algorithms such as Logistic Regression (LR), Support Vector Machines (SVM), and K-nearest neighbors (KNN) have been extensively used in student dropout prediction. A1^18^ applied AutoML, incorporating KNN and ANN, achieving strong classification accuracy. A2^23^ used Lasso-regularized LR, reaching an F1 score of 97% in online learning environments . Similarly, A3^24^ reported a 93.5% F1 score, while A4^25^ found NN models outperforming SVM and RF for assignment submission prediction.

Tree-based ensemble techniques such as Random Forest (RF), Gradient Boosting (XGB, GBoost), LightGBM, and CatBoost have consistently demonstrated superior performance across various contexts. RF was the top performer in several studies, including those by A5^26^, A6^27^, A7^28^, A8^29^, A9^30^, and A10^17^, with F1 scores ranging from 76.2% to 96%. Gradient Boosting models have also achieved high accuracy. A11^31^ reached 95.4% using academic and demographic data, while A12^32^ applied GBoost with Boruta feature selection for minimal error. A13^33^ employed XGB to predict potential dropouts across 19 schools in Brazil, achieving a recall of 72.2%. LightGBM and CatBoost demonstrated excellent results in multiple studies. A14^34^ achieved an F1 score of 84% at Sahmyook University in Seoul using academic records of 20,050 students, while A15^35^ achieved 87.4% in a South Korean distance university. A16^10^ showed LightGBM and CatBoost achieving up to 88% F1 scores with hyperparameter optimization and SMOTE resampling.

Hybrid models and deep learning techniques are increasingly employed to address data complexity and imbalance. A17^36^ used CNN-LSTM models in MOOCs, achieving 94% accuracy. A18^37^ proposed an interpretable hybrid LR–NN model with SHAP and LIME, reporting a 96% F1 score. A19^38^ introduced a CNN–Attention–XGB hybrid reaching a 97% F1 score. A20^39^ presented an OSO-DDNN model for academic performance prediction, achieving 93.8% F1 on a large-scale elementary education dataset.

Numerous studies emphasized data preprocessing, including class balancing, feature engineering and selection A3^24^, A16^10^, A18^37^, and A21^40^ also compared various resampling techniques for imbalanced data. Feature selection was another crucial aspect investigated. A12^32^ employed Boruta, RFE, and Lasso for feature selection. A10^17^ highlighted the impact of pre-processing, including outlier removal, on model performance. Explainable AI (XAI) has gained traction to improve model transparency. Tools such as SHAP and LIME were used by A18^37^, A22^41^, A23^42^, and A24^43^, confirming the role of interpretability in providing robust decision-support for educational stakeholders.

In the broader context of machine learning, the critical processes of feature selection and hyperparameter tuning have seen substantial advancements through the application of meta-heuristic optimization algorithms. Recent literature highlights the efficacy of such techniques including the Swordfish Movement, Greylag Goose, and Waterwheel Plant optimization algorithms, in solving complex, high-dimensional feature selection and forecasting problems in domains like photovoltaic power generation, wind speed forecasting, and smart city electricity load management^44–47^. While these advanced meta-heuristic approaches excel in complex time-series and continuous data environments, our study adopts tree-based feature selection techniques (RFI and RFE), which are specifically well-suited and highly interpretable for the categorical and discrete nature of educational data.

A comparative summary of selected studies is shown in Table 1, focusing on the used method, dataset, performance, and XAI Method. The evaluation of models varied across studies through performance metrics including Accuracy (Acc), Precision (Prec), Recall (Rec), F1-score (F1), and Area Under the ROC Curve (AUC). F1-score is used as the primary comparison metric due to its robustness in imbalanced classification problems. Different data splitting strategies were employed, including holdout validation with varying ratios and k-fold cross-validation. XAI Method indicates the explainable artificial intelligence technique used in each study (e.g., SHAP, LIME). ’None’ denotes that no explainability method was applied.

**Table 1:** Summary of student dropout prediction studies

Author	Year	Method	Data source	Splitting data	Performance	XAI method
A1^18^	2021	AutoML	UAE dataset	10-fold	Acc: 90%	None
A2^23^	2023	LR with Lasso	Binus University in Indonesia	holdout 70-30%	F1: 97%	None
A3^24^	2025	kNN + SMOTE	ASUPG University in Undiksha	10-fold	F1: 93.5%	None
A4^25^	2022	NN	Taiwan	-	F1: 88%	None
A5^26^	2021	RF	Philosopher University in Slovakia	10-fold	F1: 91%	None
A6^27^	2024	RF	Major university in Bangladesh	holdout 80-20%	F1: 78%	None
A7^28^	2024	RF	SATDAP in Portugal	-	F1: 76.2%	None
A8^29^	2024	RF	SATDAP in Portugal	holdout 70-30%	F1: 87.8%	None
A9^30^	2024	RF	Hons University in Malaysia	holdout 80-20%	F1: 81%	None
A10^17^	2024	RF, XGB	SATDAP in Portugal	-	F1: 96%	None
A11^31^	2024	GBoost	UNADECA data	-	Acc: 95.4%	None
A12^32^	2023	GBoost	OULA dataset	-	Acc: 78%	None
A13^33^	2023	XGB	19 schools in Brazil	-	Rec: 72.2%	None
A14^34^	2023	LightGBM	Sahmyook University in Seoul	holdout 80-20%	F1: 84%	None
A15^35^	2024	LightGBM	Distance University in South Korean	-	F1: 87.4%	None
A16^10^	2024	LightGBM, CatBoost	SATDAP in Portugal	-	F1: 88%	None
A17^36^	2024	CNN-LSTM	MOOCs in China	-	F1: 98%	None
A18^37^	2025	HLRNN model	SATDAP in Portugal	-	F1: 96%	SHAP, LIME
A19^38^	2025	CNN + Atten + XGB	SATDAP in Portugal	holdout 80-20%	F1: 97%	None
A20^39^	2024	OSO-DDNN	Elementary school	holdout 80-20%	F1: 92%	None
A21^40^	2020	Ensemble RF and SVM-SMOTE	Iran and Portugal	holdout and 5-fold	F1: 84%	None
A22^41^	2024	CatBoost	BME University in Hungary	holdout 75-25%	AUC: 77.4%	PI, PDP, LIME, SHAP
A23^42^	2025	XGB	SATDAP in Portugal	holdout 80-20%	F1: 83%	SHAP, PI
A24^43^	2025	GBoost, RF	Several institutions in Bangladesh	holdout 80-20%	F1: 86.49%	SHAP, LIME
Our	2025	Proposed SOD-FE model	SATDAP in Portugal	holdout and 5-fold	**F1: 98.09%**	SHAP
			Philosopher University in Slovakia		**F1: 98.33%**	

Significant values are in [bold].

Across the literature, tree-based ensemble models like RF, XGB, LightGBM, and CatBoost consistently demonstrate high predictive accuracy in dropout prediction tasks. Additionally, hybrid approaches and the integration of deep learning techniques are emerging as promising directions. Several pieces of literature also underscore the importance of addressing data characteristics such as class imbalance and employing appropriate evaluation metrics. However, the pre-processing stage is often neglected despite its vital role in enhancing model performance and reliability. Moreover, only a limited number of works have incorporated explainable AI (XAI) techniques to identify the most influential features and offer valuable insights for decision support systems (e.g., A18, A22, A23, A24). A further challenge lies in the limited availability and lack of public access to comprehensive educational datasets. This limitation, combined with the presence of biased or non-representative data, can result in skewed predictions, particularly if the training data disproportionately represents specific populations. The proposed SOD-FE model achieved an F1 score of 98.09% on the Portugal dataset. To ensure the generalizability of our findings, the model has also been applied to the Slovakia dataset, where the proposed model achieved an F1 score of up to 98.33%. Additionally, the SOD-FE model incorporates explainable artificial intelligence (XAI) techniques to enhance transparency and foster trust in its predictions, thereby serving as a robust decision-support tool that empowers educational stakeholders to design targeted interventions.

## Materials and methods

This study adopts the proposed SOD-FE approach to develop predictive models for the early identification of at-risk students through the use of a dataset sourced from a higher education institution. Our study focuses on optimizing the student dropout prediction by assessing the efficacy of six widely used ML classification algorithms. The proposed approach, termed SOD-FE, introduces two primary methodological contributions: supervised outlier detection (SOD) and feature engineering (FE). Structurally as shown in Fig. 1, these contributions are smoothly combined in a process that extends from obtaining the initial data acquisition to the final model selection. In particular, this section provides comprehensive coverage of every aspect of the application of data pre-processing and feature engineering methods. Each stage aims to convert the original dataset into the optimal dataset to serve as an input for classification algorithms to generate dependable predictive models for decision support systems.

### Data acquisition

The Portugal dataset was created by a higher education institution program SATDAP in Portugal between the academic years 2008/2009 to 2018/2019^48^. The Portugal dataset consists of information about students enrolled in 17 undergraduate programs, including fields such as agronomy, design, education, nursing, journalism, management, social service and technologies^49^. The Portugal dataset contains 4424 entries with 34 features along with a target variable. These features cover demographic factors, social-economic factors, and student academic factors in Table 2. The target variable classifies students into one of three categories: *enrolled* (students continuing their studies), *dropout* (students who discontinued), and *graduate* (students who completed their studies)^10^. To address the problem of student dropout prediction, we excluded the *enrolled* class from the target variable and redefined the *graduate* class as *non-dropout*. The final dataset comprises 39% *dropout* cases and 61% *non-dropout* cases (see Fig. 4c)^37^.

**Table 2:** Statistical summary and feature interpretation of the student dropout dataset

Feature	*#*Cls	Statistical distribution				Interpretation
		*μ*	*σ*	*\min*	*\max*	
Curricular Units (Approved)	-	4.0	2.9	0.0	19	Clear separation, strong predictive potential, widespread, students vary significantly in success rate and strongly associated with *dropout*
Curricular Units (Grades)	-	9.5	5.4	0.0	18	Wide ranges *μ*, high *σ*, *dropout* students skewed left, and *non-dropout* students skewed right, indicating performance disparity across the student population
Curricular Units (Evaluations)	-	7.5	3.8	0.0	25	*Dropouts* tend at low evaluations, *non-dropouts* more balanced, substantial variability (*σ*= 3.8), suggesting a diverse academic performance landscape
Curricular Units (Enrolled)	-	6.1	1.8	0.0	21.0	*Non-dropout* counts are modestly lower among *dropouts*, potentially signaling moderate relevance
Curricular Units (Credited)	-	0.3	1.5	0.0	17.0	Low mean implies crediting is rare, mostly zero-valued, *dropouts* slightly sparser, hinting at lack of progress
Curricular Units (without Eval.)	-	0.1	0.7	0.0	10.0	Mostly zeros, *non-dropout* students slightly sparser
Tuition fees up to date	2	0.8	0.4	0.0	1.0	20% late payers, unpaid tuition linked to *dropout*.
Scholarship holder	2	0.3	0.4	0.0	1.0	30% receive aid, scholarship holders are less likely to *dropout*.
Debtor	2	0.1	0.3	0.0	1.0	Correlates with *dropout*.
GDP	-	-0.1	2.2	– 4.1	3.5	No strong separation, low individual correlations, distributions similar across *dropout* and *non-dropout* groups, low direct effect
Inflation rate	-	1.3	1.4	– 0.8	3.7	
Unemployment rate	-	11.6	2.7	7.6	16.2	
Age at enrollment	-	22.9	7.4	17.0	70.0	Most in early 20s, older students more likely to *dropout* (right-skewed)
Gender	2	0.3	0.4	0.0	1.0	Balanced, low predictive value
Marital status	6	1.1	0.6	1.0	6.0	Mostly single students, certain statuses are more common among *dropout*.
Displacement status	2	0.6	0.5	0.0	1.0	Modest association with *dropout*.
Nationality	21	1.2	1.7	1.0	21.0	Mainly one nationality, some diversity
International	2	0.0	0.1	0.0	1.0	Highly sparse, slightly higher *dropout* rate
Application mode	18	6.4	5.0	1.0	17.0	Wide spread, useful categorical indicator
Application order	7	1.9	1.4	1.0	6.0	*Dropouts* clustered in lower application types
Previous qualification	17	2.1	3.4	1.0	17.0	*Dropouts* concentrated at lower qualifications
Daytime/evening attendance	2	0.9	0.3	0.0	1.0	*Dropouts* are more in evening classes
Course	17	10.4	4.1	1.0	17.0	*Dropout* rates vary by course
Educational special needs	2	0.0	0.1	0.0	1.0	Almost all coded 0, sparse, low relevance
Father’s qualification	34	16.5	11.0	1.0	34.0	Higher dropout among students with less educated parents
Mother’s qualification	34	12.6	8.9	1.0	29.0	
Father’s occupation	46	7.5	3.7	1.0	46.0	Some *dropout* clustering by occupation
Mother’s occupation	46	7.1	3.4	1.0	32.0	

**Fig. 2:**
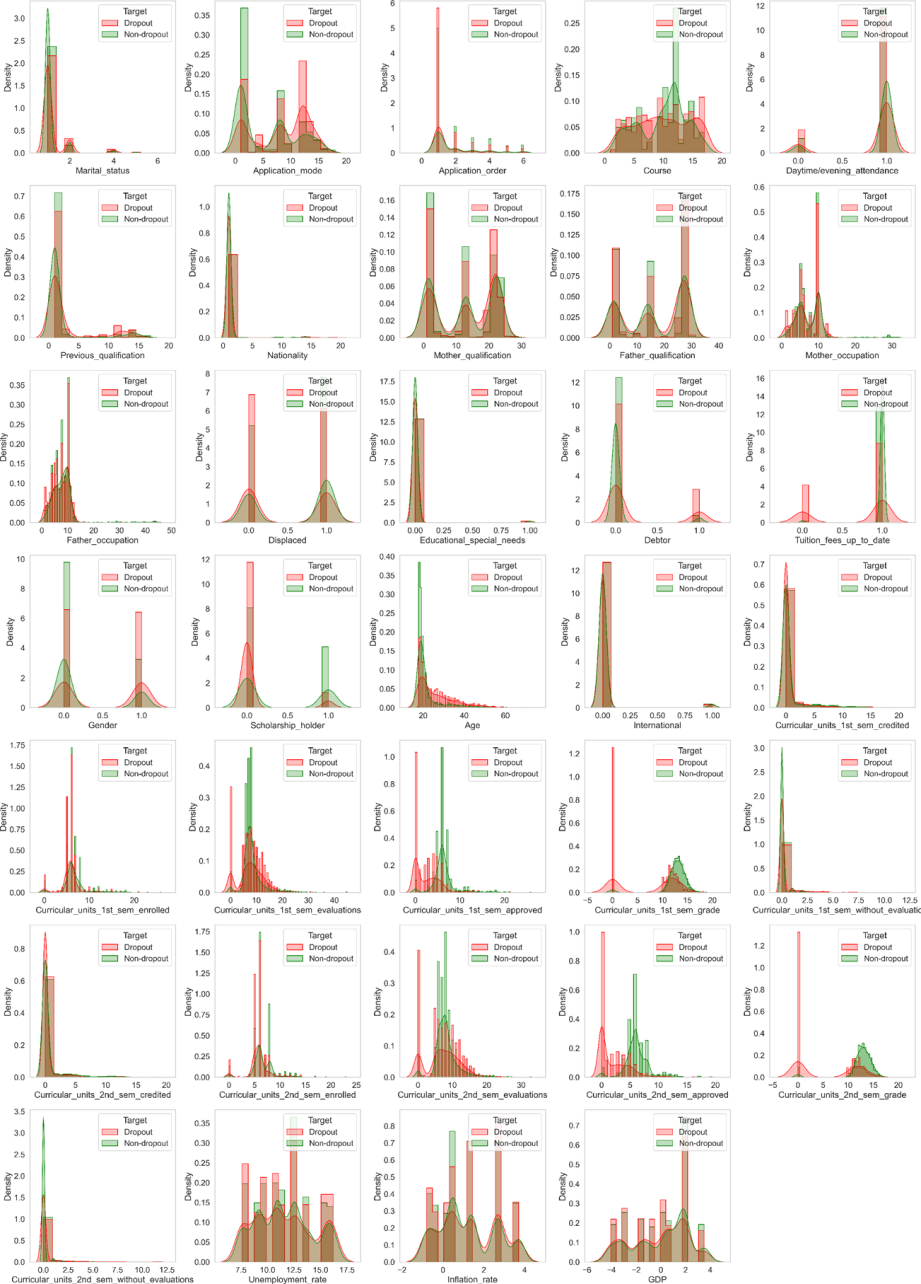
Distributional comparison of features between *Dropout* and *Non-dropout* students.

To ensure the generalizability of our study, we additionally employed a second dataset, referred to as the Slovakia dataset. This dataset was extracted from an online course hosted on the Moodle Learning Management System (LMS) at Constantine the Philosopher University in Slovakia, covering four academic years^26^. The Slovakia dataset contains 261 unique students and consists of five features that reflect their participation in various types of course-related activities.

### Data preprocessing

Data preprocessing is a critical initial stage that involves cleaning the dataset, handling missing values, removing irrelevant features, and addressing outliers to improve data quality and structure^17^. In this study, Exploratory Data Analysis (EDA) techniques were employed to identify patterns, trends, and correlations between features using statistical summaries and visualizations^38^. In addition, the proposed Supervised Outlier Detection (SOD) method was utilized during preprocessing to uncover hidden relationships between features, detect anomalous instances, and identify potential biases that may negatively influence the performance of dropout prediction models. This label-aware filtering step ensures that relevant patterns, particularly those associated with minority classes, are preserved during subsequent modeling stages.

To prepare the data for classification, it was emphasized that there were no missing values^32^. Fortunately, the missing values have previously been identified and processed appropriately. However, additional minimal cleaning procedures are needed, and they include renaming columns for a better working experience during future operations^49^.

Categorical features were converted into a numerical format via label encoding to ensure compatibility with ML algorithms, thereby preparing the dataset for further analysis. A comprehensive statistical examination was conducted to understand the distributional characteristics of the dataset, as summarized in Table 2 and Fig. 2 presents the probability density functions of all 34 features, stratified by *dropout* status. Among academic-related features such as curricular_units_approved, curricular_units_grades, and curricular_units_evaluations, distinct separability is evident. *Non-dropout* students cluster around higher values, whereas *dropout* students are concentrated in lower ranges, including zero values. This observation aligns with the summary statistics, where these features generally exhibit moderate mean values but substantial variability and skewness (e.g., curricular_units_grades: mean *≈* 9–10, standard deviation > 5), suggesting the potential need for normalization or transformation when employing models sensitive to feature scale or distributional skew.

In particular, features such as curricular_units_approved show stark contrasts, with *non-dropout* students concentrated at higher approval counts (e.g., >10), while *dropout* students are concentrated near zero, reinforcing the relationship between academic underperformance and *dropout* risk. It is crucial to distinguish between early-risk prediction and retrospective classification. The academic features utilized in this study, such as first and second-semester grades and approvals, represent early-stage indicators collected prior to the student’s final decision to drop out, thereby validating their operational use for early warning and intervention systems. Binary features, including tuition_fees, debtor, and scholarship_holder, display bimodal distributions. *Dropout* students are disproportionately represented in categories associated with financial instability (e.g., unpaid tuition), whereas *non-dropout* students dominate the opposite ends. For instance, the mean value for tuition_fees is 0.8, indicating a considerable portion of students are at financial risk. Conversely, several categorical and binary features such as nationality, international, and educational_special_needs exhibit extremely imbalanced distributions, with over 95% of values concentrated in a single category. Due to their low variance and limited discriminatory power, these features were excluded from subsequent modeling stages to reduce noise and enhance model performance. Macroeconomic indicators (GDP, unemployment_rate, and inflation_rate) present more uniform or mixed distributions, showing no strong differentiation between *dropout* and *non-dropout* students. This suggests a limited direct influence of macroeconomic conditions on individual dropout behavior. Some categorical variables, such as application_mode (18 categories) and nationality (21 categories), exhibit broad distributional variability. Although subtle, differences in categorical preferences between groups were observed, for instance, *dropout* students tend to apply earlier in the application_order, while *non-dropout* students are more evenly distributed. The age variable shows a right-skewed distribution, with most students in their early twenties. However, *dropout* students span a wider age range, including a noticeable tail extending into older age groups (e.g., over 40 years), indicating that mature students may face higher *dropout* risks. The binary target variable *dropout* exhibits a mean of 0.6 and a standard deviation of 0.5, confirming that 60% of students in the dataset discontinued their studies. This moderate class imbalance necessitates the application of resampling techniques or class-weight adjustments during model training to address potential bias and improve minority class representation. SMOTE and ADASYN were selected due to their proven effectiveness and empirical success in handling class imbalance in classification tasks. While alternative methods such as Borderline-SMOTE emphasize samples near decision boundaries, they may amplify noise in the presence of outliers. Given that the proposed SOD-FE framework already incorporates supervised outlier filtering, SMOTE and ADASYN offer a more stable and complementary approach by generating balanced and informative synthetic samples without reintroducing noisy instances.

### Supervised Outlier Detection (SOD)

The proposed approach integrates SOD with label-aware feature relevance assessment and statistical filtering. Specifically, it combines mutual information and Pearson correlation to rank features based on their association with the target variable^50^. A subset of the most informative features is then selected according to these rankings. Outlier detection is performed using an interquartile range (IQR) based statistical filter, denoted as $D(\textbf{x}_i; \alpha , \beta )$, guided by class-specific conditions *α* and the number of selected features *β*. The cleaned dataset, denoted by $D^{cl}((\textbf{x}_i, y_i); \alpha , \beta )$, is then used to train a supervised model *M*. Optimal values of *α* and *β* are selected based on the F1-score during cross-validation. This ensures that the retained features are both relevant and non-redundant, while outliers that may degrade classification performance are filtered out in a label-aware manner.

Let $\{X, Y\} = \{(\textbf{x}_i, y_i)\}_{i=1}^n$ denote a dataset of *n* labeled student records, where $\textbf{x}_i \in \mathbb {R}^d$ is a feature vector and $y_i \in \{0,1\}$ is a binary dropout label. The feature matrix *X* may be contaminated with noise or outliers. The goal is to clean *X* before training the final supervised model. The SOD method helps to determine the optimal filtering configuration.

Let the pairs $\{X_{tr}, Y_{tr}\}$ and $\{X_{ts}, Y_{ts}\}$ denote the training and testing subsets obtained via stratified splitting. The SOD method is applied exclusively to the training data matrix $X_{tr}$ to identify and filter potential outliers, resulting in a cleaned dataset $X^{cl}$ and its corresponding target vector $Y^{cl}$.

To identify the most informative features, we computed two supervised relevance scores for each feature $x_j$:

1 $$\begin{aligned} \rho _j = \textrm{corr}(x_j, y) \end{aligned}$$

2 $$\begin{aligned} \mu _j = I(x_j; y) \end{aligned}$$

Where $\rho _j$ is the absolute Pearson correlation and $\mu _j$ is the mutual information between $x_j$ and the target *y*. The combined rank for feature $x_j$ is computed as:

3 $$\begin{aligned} R_j = \frac{1}{2} \left( \textrm{rank}_{\rho }(x_j) + \textrm{rank}_{\mu }(x_j) \right) \end{aligned}$$

We select the top-*β* features with the lowest $R_j$ values, denoted by $\mathscr {F}_\beta$. IQR-based outlier detection is applied to $X_{tr}$ using only the selected feature subset $\mathscr {F}_\beta$. For each $x_j \in \mathscr {F}_\beta$, we compute:

4 $$\begin{aligned} \textrm{IQR}_j&= Q3_j - Q1_j \end{aligned}$$

5 $$\begin{aligned} \textrm{Lower}_j&= Q1_j - 1.5 \cdot \textrm{IQR}_j \end{aligned}$$

6 $$\begin{aligned} \textrm{Upper}_j&= Q3_j + 1.5 \cdot \textrm{IQR}_j \end{aligned}$$

An instance $\textbf{x}_i$ is considered an inlier if:

7 $$\begin{aligned} x_{ij} \in [\textrm{Lower}_j, \textrm{Upper}_j], \quad \forall j \in \mathscr {F}_\beta \end{aligned}$$

To address class imbalance, the outlier detection procedure is evaluated under different label conditions *(α )*. We define the detection rule $D(\textbf{x}_i; \alpha , \beta ) \in \{\text {inlier}, \text {outlier}\}$, where $\alpha \in \{0, 1, 2\}$ denotes the filtering focus: only *non-dropout* class (0), only *dropout* class (1), or *all* data (2), and *β* is the number of selected features. The cleaned training set is given by:

8 $$\begin{aligned} X_{tr}^{cl} = \left\{ \textbf{x}_i \in X_{tr} \mid D(\textbf{x}_i; \alpha , \beta ) = \text {inlier} \right\} \end{aligned}$$

To determine the optimal configuration $(\alpha ^*, \beta ^*)$, a Random Forest classifier *M* is trained and evaluated on $\{X_{tr}^{cl}, Y_{tr}^{cl}\}$ using the F1 score as the evaluation metric:

9 $$\begin{aligned} (\alpha ^*, \beta ^*) = \arg \max _{\alpha , \beta } \textrm{F1}\left( M({D}^{cl}(\alpha , \beta )) \right) \end{aligned}$$

Once the optimal hyperparameters are determined, the training and test sets are merged:

10 $$\begin{aligned} \{X, Y\} = \{X_{tr}, Y_{tr}\} \cup \{X_{ts}, Y_{ts}\} \end{aligned}$$

The final outlier detection function $D(\cdot ; \alpha ^*, \beta ^*)$ is then applied to all instances in *X* to assign a binary outlier score:

11 $$\begin{aligned} \mathrm {outlier\_score}(\textbf{x}_i) = {\left\{ \begin{array}{ll} 1 & \text {if } D(\textbf{x}_i; \alpha ^*, \beta ^*) = \text {inlier} \\ -1 & \text {if } D(\textbf{x}_i; \alpha ^*, \beta ^*) = \text {outlier} \end{array}\right. } \end{aligned}$$

This score serves as a supervised outlier indicator and is appended to the feature vector of each instance. The final dataset is:

12 $$\begin{aligned} \hat{X} = \left\{ \hat{\textbf{x}}_i = [\textbf{x}_i, \ \mathrm {outlier\_score}(\textbf{x}_i)] \right\} _{i=1}^{n} \end{aligned}$$

Where each instance $\hat{\textbf{x}}_i$ is formed by appending the supervised outlier score to the original feature vector $\textbf{x}_i$.

**Algorithm 1:**
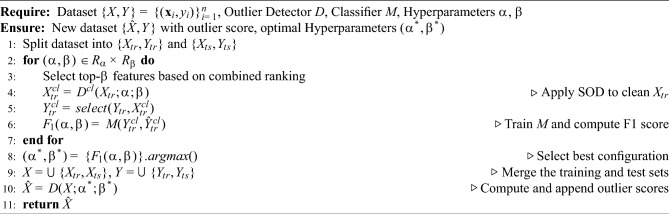
Proposed Supervised Outlier Detection (SOD) method.

The optimal hyperparameters $(\alpha ^*, \beta ^*)$ are determined via K-fold cross-validation over the search space $R_{\alpha } \times R_{\beta }$. Specifically, the parametric optimization strategy was implemented using an exhaustive Grid Search method. The search space was strictly defined as $R_\alpha = \{0, 1, 2\}$ (representing the class filtering focus) and $R_\beta = \{1, 2, \dots , d\}$ (where *d* is the maximum number of top-ranked features). Regarding computational cost, the optimization process proved to be highly efficient. Because the IQR-based filtering is univariate, its time complexity scales linearly, *O(N · d)*, with the number of instances *N* and features *d*. On our hardware setup, the complete Grid Search tuning process converged within a few minutes per fold. However, a limitation of this exhaustive grid search approach is its scalability constraint; while it is computationally feasible for two parameters (*α* and *β*), introducing additional hyperparameters in future iterations of the SOD-FE framework would exponentially increase the computational burden, thereby necessitating more scalable techniques such as Bayesian optimization or Randomized Search.

**Fig. 3:**
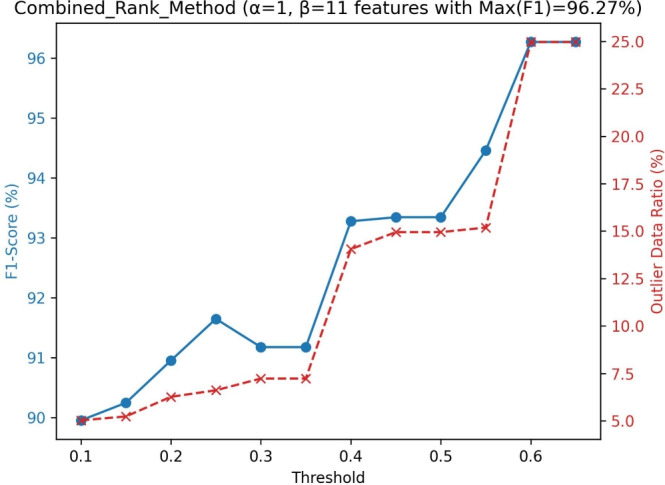
Performance of SOD with combined feature Ranking. The plot illustrates the F1 score (%) (left y-axis, blue line with circular markers) and outlier data ratio (%) (right y-axis, red dashed line with ’x’ markers) as a function of the determining the optimal hyperparameters target class ($\alpha ^*$), and number of selected features ($\beta ^*$) with the maximum F1 score.

The SOD method identifies relevant features, removes outliers and optimizes classifier performance using the F1-score as the evaluation criterion. As shown in Figure 3, this procedure enhances model robustness and generalization. The F1 score initially increases with the threshold, indicating improved model performance due to effective outlier removal. The F1 score peaks at 96.27% when the optimal hyperparameters $\alpha ^{*}=1$ and $\beta ^{*}=11$ are selected.

**Fig. 4:**
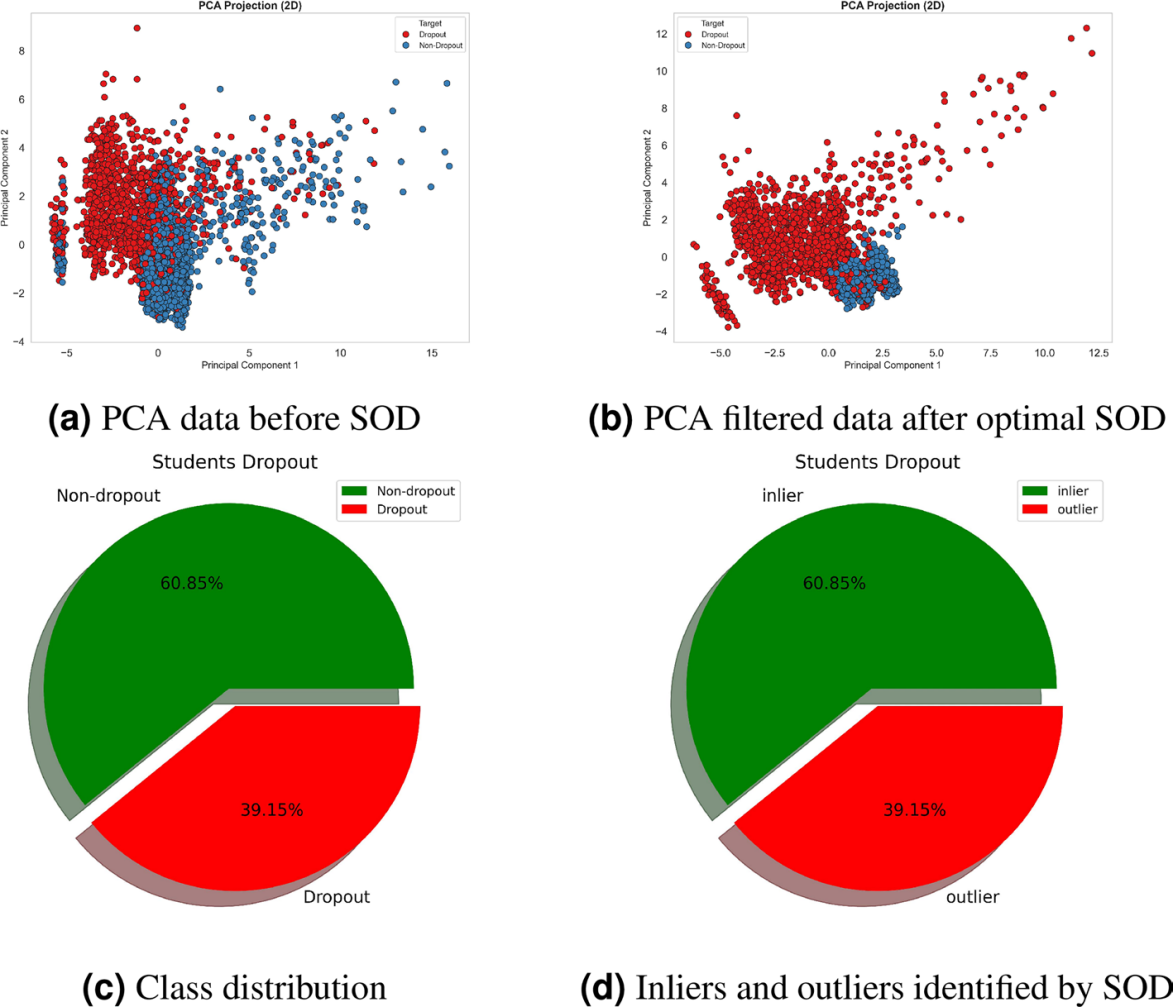
Data and class distribution used in our study.

While the SOD method applies IQR-based statistical filtering guided by Pearson correlation and label information, we acknowledge that such techniques may be limited in capturing non-linear feature interactions. To address this, we incorporated mutual information alongside Pearson correlation during feature relevance assessment, allowing the detection of both linear and non-linear dependencies. Additionally, the risk of inadvertently removing legitimate yet atypical dropout cases, such as students with unusually high or low attendance, was carefully mitigated through a parametric tuning strategy. Specifically, the outlier detection process is controlled by two parameters, *α* (class focus) and *β* (number of features), which are optimized using cross-validation to preserve patterns relevant to the minority class. This targeted adjustment ensures that difficult but valid cases are retained, rather than being misclassified as noise. Furthermore, a Component-wise Analysis section confirmed that the selected configuration achieved the best balance between noise reduction and dropout pattern preservation. The effect of applying the SOD method can be seen in Fig. 4a , b and d show the data distribution before and after the SOD-based outlier removal. These visualizations confirm the impact of the SOD method on improving data quality and model readiness.

### Feature engineering

During the feature engineering phase, the dataset receives enhancement through assessments of feature interrelationships, choosing relevant variables through apply proper transformations and feature generation techniques^22^. An extensive correlation analysis was performed to identify and discard weakly or redundant features which improved model predictive capabilities and interpretation.

#### Correlation analysis

**Fig. 5:**
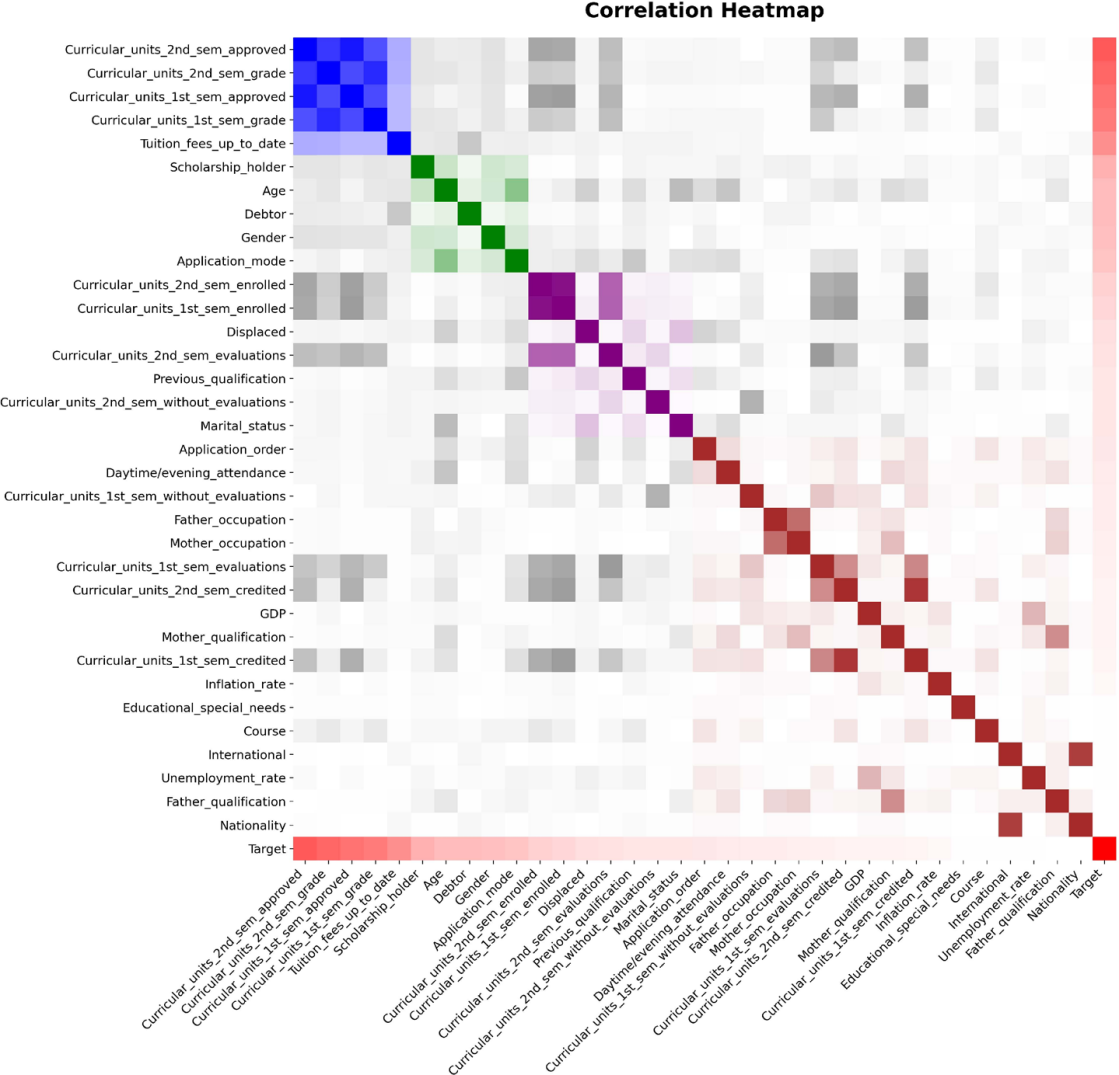
Correlation heatmap.

The Pearson correlation heatmap Fig. 5 demonstrated the relationship between all features including the target variable through a visual representation of their pairwise correlation coefficients. The heatmap uses color intensity to display linear relationship strength while light colors represent weak to nonexistent associations. Each feature shows a strong correlation with itself since the value on the diagonal is equal to 1. The visual representation allowed for the detection of multicollinearity while showing which factors are strongly linked to the target variable. The analysis implemented absolute values of correlation coefficient measurements to study linear associations with positive or negative mathematical relationships. The absolute correlations between features and the target variable are classified into strong (*łe* 0.4), moderate (*łe* 0.2 and < 0.4), weak (*łe* 0.1 and < 0.2) and negligible (< 0.1) categories. A specific color scheme distinguished each group in the heatmap (blue, green, purple, and brown respectively) while red indicated the target variable.

Academic performance-related features displayed powerful relationships with the target during the analysis which indicated their strong predictive potential. The combination of financial indicators with other features or their application in non-linear models can derive additional predictive value despite their moderate and weak correlations. A comprehensive analysis should evaluate the combined impact of macroeconomic indicators on high school dropout rates even though each metric measured separately has minimal individual correlation. Educational_special_needs, international and nationality features displayed minimal correlation with student dropout instead of enhancing prediction accuracy therefore these features could safely be omitted to reduce model complexity and overfitting risk.

#### Random Forest Importance (RFI)

**Fig. 6:**
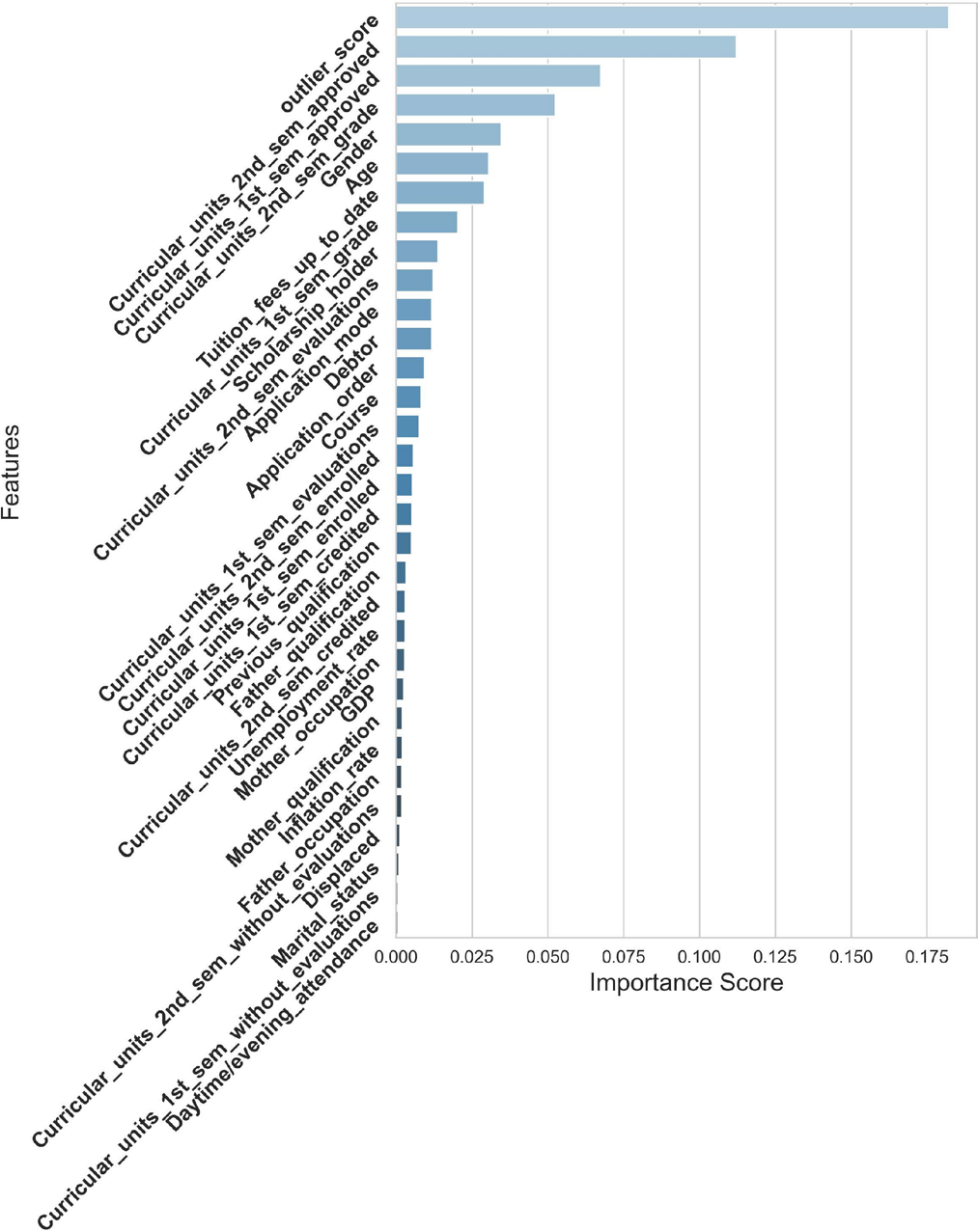
Feature importance analysis using the RFI method.

RFI techniques evaluated the importance of features through a ranking system for features. As shown in Figure 6. To find out the most important features, we applied the test using F1 score as the error measure, which works better for cases where the data are not balanced^49^. This result enabled the recognition of non-essential features which proved either insignificant or unimportant for the prediction model. The computational efficiency and generalizability were enhanced by excluding these features. This feature selection process allows the model to train using only important variables, thereby achieving high accuracy, maintaining robustness, and preventing overfitting caused by irrelevant inputs^51^. The top twenty-five features representing demographic, academic, and socio-economic factors were chosen from the initial dataset. These selected features are further discussed and explained in the Model Interpretability section.

#### Recursive Feature Elimination (RFE)

The RFE technique functions as a ML procedure that performs repeated eliminations of unimportant features from datasets to achieve better model performance. RFE approach assigns weights to each feature for its predictive power in determining the target variable before removing the least important features successive times until the specified number of features remains, while considering their impact on the output variable prediction. RFE serves as an enhancement tool for other ML methods to boost their operational efficiency^32^. The proposed model displays different performance results with the inclusion of the twenty-eight most important features. With the previous twenty-five features, the Previous_qualification, Inflation_rate and GDP features were selected. This inclusion emphasizes the important role that economic factors and broader environmental conditions can play in influencing students’ motivation and ability to complete their studies. The RFI method was adopted to evaluate our proposed SOD-FE approach for predicting students academic success. The impact of employing both the RFI and RFE feature selection methods is further discussed in the Feature Selection Analysis section.

### Data normalization

Data normalization or feature scaling was performed before starting to model building stage. This is important because most ML models depend on Euclidean distance which may not work well unless features are scaled properly. The standardization method was used to make the model more efficient. All values were adjusted so they behaved as a standard normal distribution, having a mean (*μ*) of 0 and a standard deviation (*σ*) of 1. The scaling is performed using the following formula:

13 $$\begin{aligned} z = \frac{x - \mu }{\sigma } \end{aligned}$$

Where *x* represents the existing value of the feature, *μ* is the average of the feature, and *σ* represents the standard deviation. As a result, every standardized feature will be centered at 0 and will have a standard deviation of 1.

### Data splitting

The normalized dataset is divided into training and testing subsets to ensure balanced label distributions and prevent bias in model evaluation. This study employs two widely used cross-validation strategies: random hold-out and shuffled k-fold cross-validation. Initially, randomly 80% of the total dataset is included in the training set and the remaining 20% is assigned to the test set. To obtain more reliable and generalizable results, we also apply 5-fold cross-validation. This method ensures the model performs well on unseen data by systematically splitting the dataset into five equal subsets. In every iteration, a portion of the data is used for testing and the rest for training. To strictly prevent data leakage, the SOD-FE pipeline was structured such that all preprocessing parameters, including the optimal *α* and *β* thresholds for outlier detection, feature selection rankings, and scaling metrics, were computed exclusively within the training folds and subsequently applied to the validation/test folds without modification.

### Machine Learning (ML) methods

This paper focuses on LR, SVM, RF, XGB, and MLP models to analyze factors affecting and predict student performance. The selected methods encompass both traditional and ensemble techniques, enabling robust comparative evaluation. The ensemble method or ensemble learning is a model that makes predictions based on the number of different models. The goal of ensemble learning is to combine the predictions made by several estimators built using a particular algorithm so that they can produce better and more effective results. The two families of ensemble methods are generally known as averaging and boosting methods. When using averaging, different estimators are built first and then their estimates are averaged. As a result, the combined estimator has a lower variance and usually performs better than any of the original estimators, e.g. RF method. Boosting methods involve creating base estimators one after another and attempting to reduce the bias in the final Averages learning with Genetic Algorithms. The goal is to use a number of weak models to make a more powerful group model, such as XGB^52^.

#### Logistic Regression (LR)

The LR model is a widely used statistical and ML algorithm for binary classification problems. To assign an input to a specific class, this algorithm takes a logistic function of a linear combination of the input features. The logistic function (sigmoid) transforms values into a range between 0 and 1, which can be interpreted as probabilities, and based on a chosen threshold (typically 0.5), the model assigns the input to one of the two categories. Its simplicity, interpretability, and effectiveness in handling categorical outcomes make it widely used in fields such as healthcare, finance, and social sciences^53^.

#### Support Vector Machine (SVM)

The SVM classifies with a hyperplane that defines classes in the feature space so that there is a large margin between them. The support vectors are the points closest to the hyperplane where the decision is made. Considering that SVM uses kernel tricks, it is effective for both high-dimensional inputs and sparse data^54^. The algorithm has demonstrated strong performance in classification tasks, including predicting student dropout rates and achieving high precision, accuracy, and sensitivity^18^.

#### Random Forest (RF)

The RF model is an ensemble learning method that uses the bagging technique by generating bootstrap samples to build multiple decision trees during training to enhance predictive accuracy and reduce overfitting. For classification, the algorithm outputs the mode of prediction, while for regression, it calculates the mean, demonstrating its versatility across different problem types. It achieves diversity among trees by selecting random subsets of features and data points, making it well-suited for handling complex large datasets with high dimensionality. Additionally, RF provides insights into feature importance, helping users identify key features that influence predictions and improving model interpretability^55^.

#### Extreme Gradient Boosting (XGB)

The XGB model works well and is easy to use in different contexts. It works by sequentially producing multiple decision trees, while each new tree tries to correct mistakes made by the previous ones. XGB relies on a Taylor expansion of the loss function. It also includes a regularization to prevent overfitting often leading to high predictive power and scalability in various ML tasks^56^.

#### Multi-Layer Perceptron (MLP)

The MLP model is an artificial neural network with several layers that are all connected. Usually, a neural network includes an input layer, one or several hidden layers, and an output layer. MLP uses a non-linear activation function in its hidden layers to understand difficult patterns between what is input and the output. The network is trained using backpropagation to adjust the weights of the connections between neurons^57^.

### Performance evaluation

The reliability of model results is verified through performance metrics including **accuracy**, **precision**, **recall**, **F1 score**, and **ROC-AUC**, as well as confusion matrices. As the most widely used performance metric, **accuracy** determines the ratio of successful predictions in relation to the total number of samples. This metric is most effective when applied to datasets with relatively balanced class distributions. Both **precision** and **recall** provide greater insight than accuracy when analyzing unevenly distributed data. The **F1 score** is a crucial metric for evaluating model performance, especially in the context of imbalanced datasets. It represents the harmonic mean of precision and recall, offering a unified measure of performance.

The sequence of Eqs. (14)–(17) defines the computations for these performance metrics:

14 $$\begin{aligned} \text {Accuracy} = \frac{TP + TN}{TP + TN + FP + FN} \end{aligned}$$

15 $$\begin{aligned} \text {Precision} = \frac{TP}{TP + FP} \end{aligned}$$

16 $$\begin{aligned} \text {Recall} = \frac{TP}{TP + FN} \end{aligned}$$

17 $$\begin{aligned} \text {F1}\_\text {score} = 2 \times \frac{\text {Precision} \times \text {Recall}}{\text {Precision} + \text {Recall}} \end{aligned}$$

ROC-AUC is commonly relied upon as a diagnostic metric for binary classification tasks. It calculates the difference between how much the model identifies both the positive and the negative classes. It shows a graph of TPR against FPR by using various threshold values. All scores in evaluation are between 0 and 1, and higher numbers show that the performance is stronger.

## Results

An experimental validation process was carried out to test the SOD-FE approach for student academic success prediction and dropout risk assessment. The Python 3.9 experimental pipeline included Pandas, NumPy, and Scikit-learn among other essential libraries. A Windows 11 workstation with dual Intel Core I7 CPUs (2.9 GHz) running 16 GB of RAM served as the platform for all testing activities. The dataset exists under ”Dataframe” and serves as the primary data format for our entire research process. Table 3 presents the ML models and their hyperparameter settings used in this paper.

**Table 3:** Hyperparameters of machine learning (ML) methods used in our study

Method	Hyperparameters
LR	C=100, penalty=’l2’, solver=’lbfgs’, max_iter=1000
SVM	kernel=’linear’
RF	n_estimators=100, min_samples_leaf=2
XGB	’n_estimators’=500, scale_pos_weight=0.82, ’max_depth’=8, ’gamma’=0.8, ’learning_rate’=0.05
MLP	activation=’relu’, solver=’adam’, max_iter=500, learning_rate_init=0.001, early_stopping=True

Our results in Tables 4 and 5 demonstrate the significant impact of SOD-FE on classifier performance. Across both hold-out and 5-fold cross-validation strategies, and on Portugal and Slovakia datasets, SOD-FE consistently improved accuracy, F1 Score, and AUC for Logistic Regression, SVM, Random Forest, XGB, and MLP models. Specifically, when analyzing the hold-out validation results presented in Table 4, without SOD-FE, AUC values ranged from 89.66% to 99.00%. With SOD-FE, the RF and XGB classifiers demonstrated the most substantial improvements. For example, RF accuracy increased from 90.08% to 98.02% and AUC from 94.55% to 99.43% on the Portugal dataset with SOD-FE+SMOTE. Similarly, XGB showed an increase in F1 Score from 85.87% to 97.72% on the Portugal dataset with SOD-FE+SMOTE. Notably, the MLP performance on the Slovakia dataset improved marginally or even decreased slightly in terms of F1 Score and AUC (from 83.87% to 84.62% and from 97.86% to 97.34%, respectively, with SOD-FE+SMOTE). This may reflect the sensitivity of neural networks to feature transformations when data size or quality is variable. The tables also include performance with SOD-FE+ADASYN, which generally showed strong results comparable to SOD-FE+SMOTE, with RF and XGB reaching 100% AUC on the Slovakia dataset.

**Table 4:** Performance of the classifiers based on the hold-out strategy on SOD-FE with SMOTE and ADASYN balanced datasets.

Model	Dataset	Without SOD-FE			With SOD-FE+SMOTE			With SOD-FE+ADASYN		
		Accuracy	F1 Score	AUC	Accuracy	F1 Score	AUC	Accuracy	F1 Score	AUC
LR	Portugal	90.08	87.67	**95.56**	96.15	96.15	99.43	95.24	95.24	99.01
	Slovakia	**96.88**	**92.86**	97.71	95.79	95.74	98.51	94.79	94.77	99.12
SVM	Portugal	89.26	86.91	95.45	96.85	96.85	99.40	95.13	95.12	98.95
	Slovakia	95.31	89.66	96.14	95.79	95.74	98.23	94.79	94.77	98.59
RF	Portugal	90.08	87.32	94.55	**98.25**	**98.25**	99.55	97.80	97.79	99.48
	Slovakia	**96.88**	**92.86**	**98.71**	95.79	95.74	**99.72**	**100.00**	**100.00**	**100.00**
XGB	Portugal	88.98	85.87	94.38	97.78	97.78	**99.67**	**98.14**	**98.14**	**99.64**
	Slovakia	95.31	89.66	99.00	**97.89**	**97.88**	99.58	**100.00**	**100.00**	**100.00**
MLP	Portugal	**91.18**	**89.00**	95.27	96.15	96.15	99.41	95.59	95.58	99.09
	Slovakia	92.19	83.87	97.86	87.37	87.47	97.34	82.29	82.36	91.67

Significant values are in [bold].

To assess the robustness and generalizability of the models, shuffle 5-fold cross-validation was conducted (Table 5). The standard deviations reported indicate stability in model performance. Again, SOD-FE yielded consistent improvements across most models and metrics. For instance, RF on the Portugal dataset improved from an accuracy of 90.74±1.3% to 98.09±0.6% with SOD-FE+SMOTE, and XGB from 90.63±0.9% to 97.83±0.5% with SOD-FE+SMOTE. The cross-validation results further solidify the efficacy of SOD-FE, demonstrating its ability to enhance model performance across different balancing strategies and provide more generalized results.

**Table 5:** Performance of the classifiers based on the shuffle 5-fold cross-validation on SOD-FE with SMOTE and ADASYN balanced datasets.

Model	Dataset	Without SOD-FE			With SOD-FE+SMOTE			With SOD-FE+ADASYN		
		Accuracy	F1 Score	AUC	Accuracy	F1 Score	AUC	Accuracy	F1 Score	AUC
LR	Portugal	91.07±1.1%	90.98±1.1%	**95.39±0.7%**	96.69±0.7%	96.68±0.7%	99.51±0.1%	94.71±1.1%	94.70±1.1%	98.85±0.2%
	Slovakia	95.61±0.6%	95.53±0.6%	97.87±1.2%	96.41±1.8%	96.40±1.9%	99.15±0.6%	94.15±2.2%	94.13±2.2%	98.31±1.1%
SVM	Portugal	**91.13±0.9%**	**91.01±0.9%**	95.15±0.7%	96.80±0.6%	96.80±0.6%	99.51±0.1%	94.92±1.1%	94.91±1.1%	98.81±0.2%
	Slovakia	**95.61±1.2%**	**95.56±1.2%**	**98.11±1.2%**	**97.04±1.8%**	**97.04±1.8%**	99.09±0.7%	94.36±1.4%	94.34±1.4%	98.42±1.0%
RF	Portugal	90.74±1.3%	90.63±1.4%	95.22±1.0%	**98.09±0.6%**	**98.09±0.6%**	**99.58±0.1%**	**97.82±0.6%**	**97.82±0.6%**	**99.47±0.2%**
	Slovakia	94.99±1.5%	94.96±1.5%	98.23±1.5%	96.62±1.6%	96.62±1.6%	**99.67±0.4%**	**98.32±1.4%**	**98.33±1.4%**	**99.86±0.2%**
XGB	Portugal	90.63±0.9%	90.55±0.9%	94.82±1.0%	97.83±0.5%	97.83±0.5%	99.57±0.2%	97.79±0.5%	97.79±0.5%	99.61±0.2%
	Slovakia	94.67±1.2%	94.63±1.3%	97.17±1.9%	95.35±2.6%	95.36±2.6%	98.59±0.9%	97.28±2.0%	97.28±2.0%	99.78±0.2%
MLP	Portugal	90.85±1.0%	90.74±1.0%	94.80±0.8%	96.99±0.5%	96.99±0.5%	99.57±0.1%	94.57±0.8%	94.56±0.8%	98.88±0.2%
	Slovakia	93.72±1.8%	93.44±1.9%	96.94±3.7%	85.42±6.0%	85.26±6.2%	97.85±0.7%	88.94±8.2%	88.94±8.1%	94.90±5.2%

Significant values are in [bold].

**Fig. 7:**
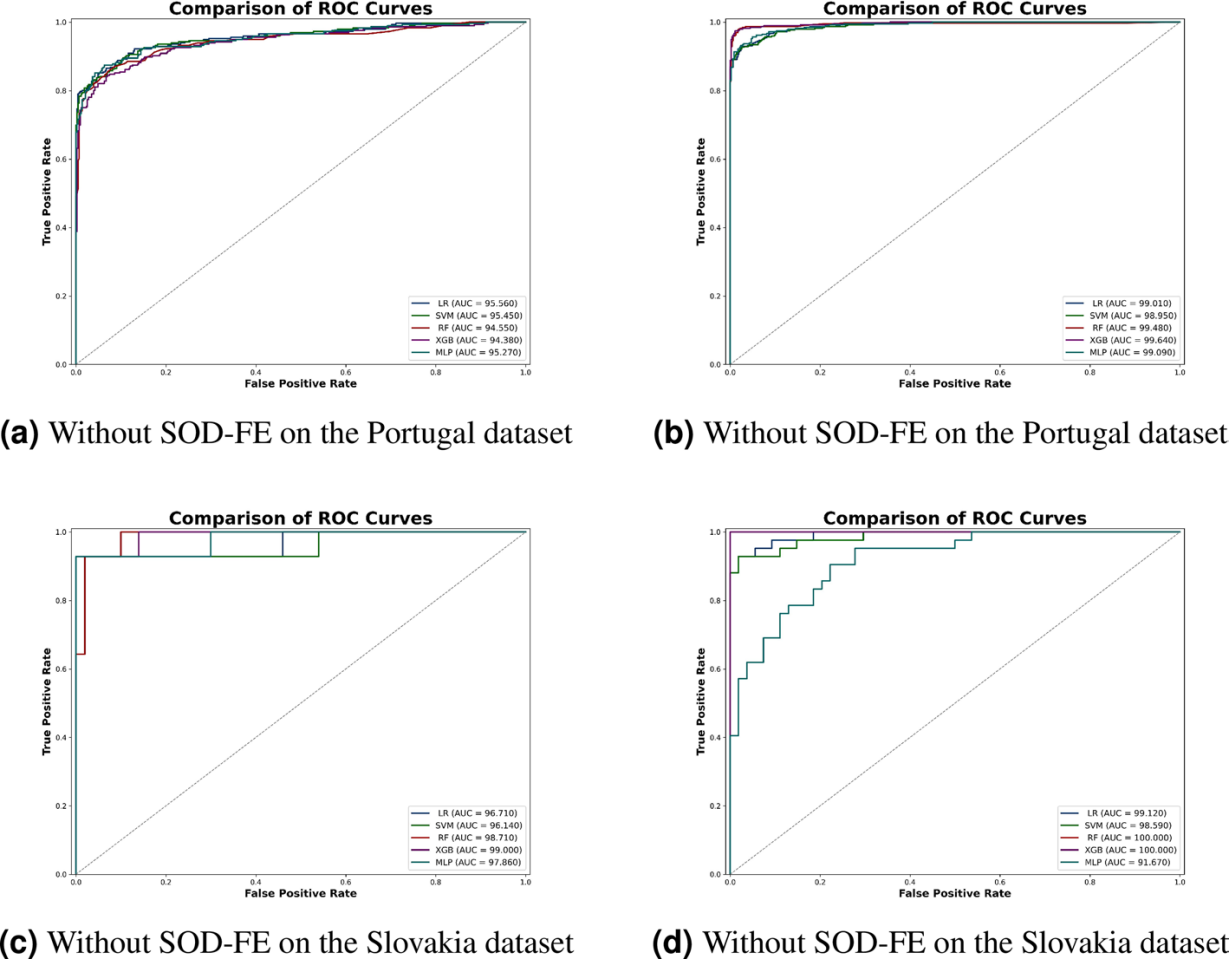
ROC curve comparison for classifiers on Portugal and Slovakia datasets, before and after SOD-FE integration.

This clear enhancement in AUC, as visualized in the ROC curves in Fig. 7, specifically comparing the “Without SOD-FE” panels (7a and c for Portugal and Slovakia datasets respectively) against the implied “With SOD-FE” scenarios from the overall performance improvements in Fig. 7b and d. It suggests that SOD-FE significantly improves the models’ ability to distinguish between classes, leading to better overall performance and robustness. The consistently higher true positive rates at lower false positive rates in the “With SOD-FE” cases (as discussed in the context of the tables) directly correlate with the shift of the ROC curves towards the top-left corner. The reduced variance in performance metrics observed with SOD-FE, especially in the cross-validation results, further indicates the stability and reliability of the enhanced feature set. This demonstrates that SOD-FE effectively captures underlying patterns, leading to more generalizable models.

**Fig. 8:**
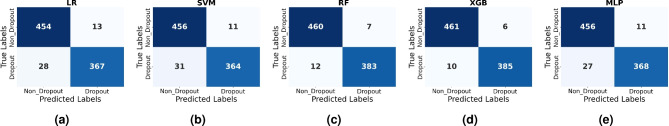
Confusion matrices of ML models for dropout prediction on the Portugal dataset.

**Fig. 9:**
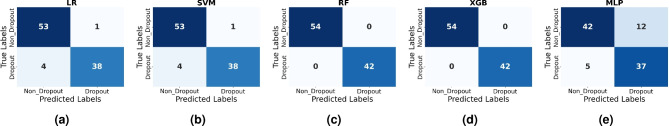
Confusion matrices of ML models for dropout prediction on the Slovakia dataset.

The confusion matrices, presented in Fig. 8 for the Portugal dataset and Fig. 9 for the Slovakia dataset, provide detailed insights into the prediction results and misclassifications for *dropout* and *non-dropout* students across the evaluated ML models. On the Portugal dataset (Fig. 8), the RF and XGB models demonstrated strong performance. The RF classifier correctly identified 460 *non-dropout* students and 383 *dropout* students, with only 7 false positives and 12 false negatives. XGB showed similar efficacy, correctly predicting 461 *non-dropout* students and 385 *dropout* students, with 6 false positives and 10 false negatives. LR also exhibited high reliability, with 454 correct predictions for *non-dropout* students and 367 for *dropout* students, resulting in 13 false positives and 20 false negatives. SVM and MLP maintained low misclassification rates. SVM correctly identified 456 *non-dropout* and 364 *dropout* students, with 11 false positives and 31 false negatives. MLP showed 456 correct *non-dropout* predictions and 363 correct *dropout* predictions, with 11 false positives and 27 false negatives.

On the Slovakia dataset (Fig. 9), the RF and XGB models achieved remarkably high accuracy, both correctly identifying 54 *non-dropout* students and 42 *dropout* students, with zero false positives and zero false negatives. LR also performed very well, correctly predicting 53 *non-dropout* students and 38 *dropout* students, with only 1 false positive and four false negatives. SVM showed similar robust performance, correctly identifying 53 *non-dropout* students and 38 *dropout* students, with one false positive and four false negatives. MLP, while still effective, had a slightly higher number of misclassifications, correctly predicting 42 *non-dropout* students and 37 *dropout* students, with 12 false positives and 5 false negatives. These results validate the ensemble-based classifiers (RF and XGB) as highly effective solutions for identifying students at risk of dropping out, as they consistently minimize both false positive and false negative errors across both datasets.

Figures 8 and 9 show that most classification models achieve strong accuracy when predicting *non-dropout* students. False negatives that predict students to remain enrolled while they leave school represent crucial concerns for educational applications because they prevent important intervention efforts. The RF and XGB classifiers demonstrated the lowest false negative rate which demonstrates their strong potential for operational early warning systems, provided that the requisite data infrastructure and computational resources are available.

### Model Interpretability

**Fig. 10:**
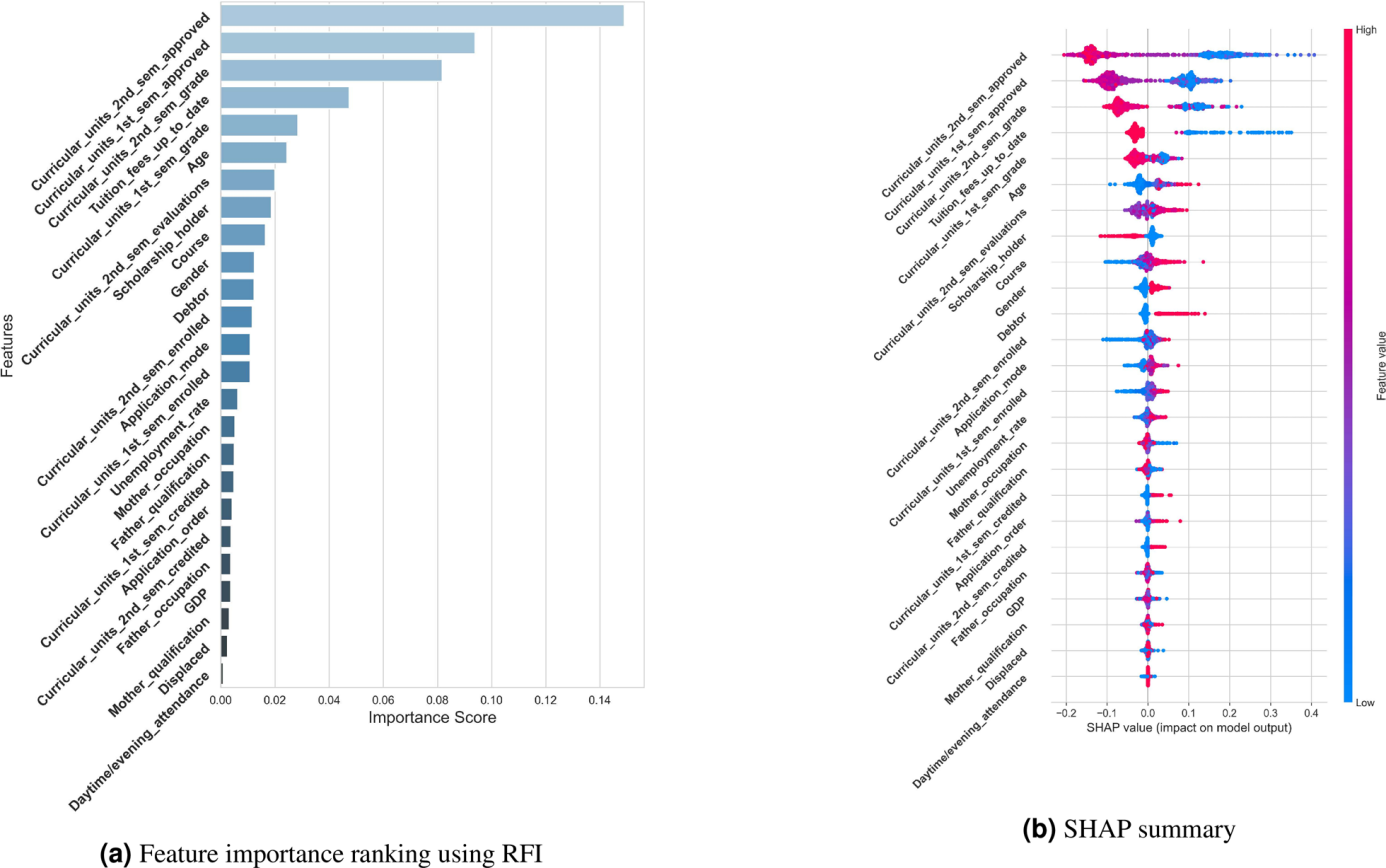
Comparison of Global and Local Feature Importance analysis using the RFI and SHAP methods.

This study employed a combination of feature ranking and explainability techniques, including correlation heatmap, RFI, and SHAP to improve predictive model interpretability while finding influential features that lead to student dropout. The correlation and RFI offer global insights into feature importance and help understand model effects but they lack the ability to explain single prediction outcomes. In contrast, the SHAP creates interpretability on both a global and local scale by providing numerical values, allowing stakeholders to assess the impact of each feature on a specific prediction outcome. This capability enables not only transparency and model auditing before deployment, but also actionable insight into student-level risk factors. Figure 10 shows an assessment of important features via RFI (Fig. 10a) compared against the results obtained from SHAP (Fig. 10b). For example, SHAP visualizations revealed that students with a high number of curricular_units_approved are significantly less likely to drop out, reinforcing educational theories that associate academic achievement with retention. Other influential features identified through SHAP include gender, age, tuition_fees, debtor, unemployment_rate, and scholarship_holder, emphasizing the role of both demographic and financial factors.

The SHAP method surpasses other methods because it demonstrates how each unique situation responds to different feature inputs. For example, a higher number of curricular_units_approved proves to be an extremely effective indicator for lower dropout risk. The results support educational theory that academic excellence during previous semesters leads students to persist in their education. Younger students and the Female exhibit higher continued enrollment probabilities according to the SHAP feature analysis. Students who maintain stable financial conditions, which include scholarship_holder status and not identified as a debtor tend to achieve greater academic success. Predictive socioeconomic factors like Mother_qualification and Father_occupation revealed lower positions on the plot and a small impact on prediction results. RFI provides insufficient detail to reveal this degree of information. SHAP analysis grants increased insight through visual representations that display how features affect each particular case. Such insights can guide targeted interventions like financial aid counseling or academic mentoring. In addition to global feature importance, local SHAP explanations were utilized for misclassified instances (false positives and false negatives). The analysis revealed that atypical combinations such as students having high financial debt despite maintaining good academic grades, occasionally misled the classifier, highlighting the nuanced reality of student dropout.

### Feature selection analysis

This subsection analyzes the impact of feature selection methods, Random Forest Importance (RFI) and Recursive Feature Elimination (RFE) on the performance of various machine learning models, compared to using all features, on the Portugal dataset. The analysis in Table 6 demonstrates that both RFI and RFE feature selection methods produce comparable high-performing models, with minor differences between them. RF showed the best overall performance, with RFI- selected features yielding slightly higher accuracy and F1 score (98.09%) and AUC (99.58%) compared to RFE-selected features (98.02% accuracy/F1, 99.57% AUC) and the baseline (97.78% accuracy/F1, 99.50% AUC). XGB performed exceptionally well even without feature selection, achieving the highest AUC (99.66%), though RFE also resulted in a strong AUC (99.62%). Multilayer Perceptron (MLP) performance was positively impacted by RFI, showing an improved F1 score (96.99%) and AUC (99.57%) over the baseline (96.64% F1, 99.51% AUC), but RFE led to a slight decrease in performance compared to RFI. SVM and LR models showed robust and consistent high performance across all three feature sets, indicating their less sensitivity to feature selection methods.

**Table 6:** Performance comparison of models using without-selected, RFI-selected, and RFE-selected features on the Portugal dataset.

Model	Without-selected features			RFI-selected features			RFE-selected features		
	Accuracy	F1 Score	AUC	Accuracy	F1 Score	AUC	Accuracy	F1 Score	AUC
LR	96.62	96.61	99.50	96.69	96.68	99.51	96.50	96.50	99.49
SVM	96.90	96.89	99.50	96.80	96.80	99.51	96.87	96.87	99.49
RF	**97.78**	**97.78**	99.50	**98.09**	**98.09**	**99.58**	**98.02**	**98.02**	99.57
XGB	97.71	97.71	**99.66**	97.83	97.83	99.57	97.83	97.83	**99.62**
MLP	96.64	96.64	99.51	96.99	96.99	99.57	96.71	96.71	99.45

Significant values are in [bold].

The feature engineering strategy employed in this study combined correlation analysis, Random Forest Importance (RFI), and Recursive Feature Elimination (RFE). These methods were chosen due to their proven efficiency in identifying relevant and non-redundant features in classification tasks, particularly in educational data mining contexts. The combination provided a structured approach for dimensionality reduction and interpretability. However, this approach is not without limitations. Correlation analysis primarily captures linear dependencies, which may not fully reflect the complex, non-linear interactions often present in dropout-related variables. Similarly, RFE can be sensitive to the initial feature set, potentially leading to suboptimal subsets, especially when informative features only become significant in interaction with others. While the integration of these methods resulted in modest performance improvements, the gains were not substantial across all models. Therefore, we recommend further exploration of non-linear feature selection techniques, such as embedded deep learning-based selection, to better capture complex relationships in educational datasets. Future work may also benefit from incorporating feature construction based on domain knowledge, and conducting ablation studies to more rigorously assess the contribution of each feature to model performance.

### Component-wise analysis

This subsection mainly discusses the effects of the component-wise performance analysis of the SOD-FE approach (including supervised outlier detection (SOD) and feature engineering (FE)) and balancing techniques (including SMOTE and ADASYN). Tables 7 and 8 present a detailed ablation study using the RF classifier, examining the impact of different data preprocessing and feature engineering strategies on student dropout prediction in the Portugal and Slovakia datasets, respectively. The performance is evaluated using standards like accuracy, precision, recall, F1 score, and AUC based on the 5-fold cross-validation.

**Table 7:** Comparison of performance metrics of SOD-FE with Random Forest classifier on the Portugal dataset when using different components

SMOTE	ADASYN	SOD	FE	Accuracy	Precision	Recall	F1	AUC
				90.74	90.91	90.74	90.63	95.22
*\checkmark*				90.74−	90.91−	90.74−	90.63−	95.22−
	*\checkmark*			90.74−	90.91−	90.74−	90.63−	95.22−
		*\checkmark*		97.99$\boldsymbol{\uparrow }$	98.01$\boldsymbol{\uparrow }$	97.99$\boldsymbol{\uparrow }$	97.98$\boldsymbol{\uparrow }$	99.21$\boldsymbol{\uparrow }$
*\checkmark*		*\checkmark*		97.78$\boldsymbol{\downarrow }$	97.81$\boldsymbol{\downarrow }$	97.78$\boldsymbol{\downarrow }$	97.78$\boldsymbol{\downarrow }$	99.50$\boldsymbol{\uparrow }$
	*\checkmark*	*\checkmark*		97.54$\boldsymbol{\downarrow }$	97.56$\boldsymbol{\downarrow }$	97.54$\boldsymbol{\downarrow }$	97.54$\boldsymbol{\downarrow }$	99.51$\boldsymbol{\uparrow }$
		*\checkmark*	*\checkmark*	98.07$\boldsymbol{\uparrow }$	98.09$\boldsymbol{\uparrow }$	98.07$\boldsymbol{\uparrow }$	98.07$\boldsymbol{\uparrow }$	99.26$\boldsymbol{\uparrow }$
	*\checkmark*	*\checkmark*	*\checkmark*	97.72$\boldsymbol{\downarrow }$	97.74$\boldsymbol{\downarrow }$	97.72$\boldsymbol{\downarrow }$	97.72$\boldsymbol{\downarrow }$	99.48$\boldsymbol{\uparrow }$
*\checkmark*		*\checkmark*	*\checkmark*	**98.09** $\boldsymbol{\uparrow }$	**98.10** $\boldsymbol{\uparrow }$	**98.09** $\boldsymbol{\uparrow }$	**98.09** $\boldsymbol{\uparrow }$	**99.58** $\boldsymbol{\uparrow }$

Significant values are in [bold].

According to the results in Tables 7 and 8, the proposed method gave the best accuracy, precision, recall, F1 score, and AUC on both datasets.For instance, on the Portugal dataset, combining SOD and FE with SMOTE achieved an F1-Score of 98.09%, compared to baselines of around 90.63%. Similarly, for the Slovakia dataset, the combined SOD-FE with ADASYN reached an F1-Score of 98.33%, demonstrating superior performance over baselines around 94.96%. Furthermore, the choice of balancing technique also influences performance.

**Table 8:** Comparison of performance metrics of SOD-FE with Random Forest classifier on the Slovakia dataset when using different components

SMOTE	ADASYN	SOD	FE	Accuracy	Precision	Recall	F1 Score	AUC
				94.99	95.12	94.99	94.96	98.23
*\checkmark*				96.88$\boldsymbol{\uparrow }$	96.88$\boldsymbol{\uparrow }$	96.88$\boldsymbol{\uparrow }$	96.88$\boldsymbol{\uparrow }$	98.71$\boldsymbol{\uparrow }$
	*\checkmark*			96.88$\boldsymbol{\uparrow }$	96.88$\boldsymbol{\uparrow }$	96.88$\boldsymbol{\uparrow }$	96.88$\boldsymbol{\uparrow }$	99.14$\boldsymbol{\uparrow }$
		*\checkmark*		95.62$\boldsymbol{\uparrow }$	95.71$\boldsymbol{\uparrow }$	95.62$\boldsymbol{\uparrow }$	95.53$\boldsymbol{\uparrow }$	99.26$\boldsymbol{\uparrow }$
*\checkmark*		*\checkmark*		96.62$\boldsymbol{\uparrow }$	96.66$\boldsymbol{\uparrow }$	96.62$\boldsymbol{\uparrow }$	96.62$\boldsymbol{\uparrow }$	99.67$\boldsymbol{\uparrow }$
	*\checkmark*	*\checkmark*		98.32$\boldsymbol{\uparrow }$	98.42$\boldsymbol{\uparrow }$	98.32$\boldsymbol{\uparrow }$	98.33$\boldsymbol{\uparrow }$	99.86$\boldsymbol{\uparrow }$
		*\checkmark*	*\checkmark*	95.62−	95.71−	95.62−	95.53−	99.26−
*\checkmark*		*\checkmark*	*\checkmark*	96.62−	96.66$\boldsymbol{\uparrow }$	96.62$\boldsymbol{\uparrow }$	96.62$\boldsymbol{\uparrow }$	99.67$\boldsymbol{\uparrow }$
	*\checkmark*	*\checkmark*	*\checkmark*	**98.32** $\boldsymbol{\uparrow }$	**98.42** $\boldsymbol{\uparrow }$	**98.32** $\boldsymbol{\uparrow }$	**98.33** $\boldsymbol{\uparrow }$	**99.86** $\boldsymbol{\uparrow }$

Significant values are in [bold].

As observed in the component-wise analysis, integrating multiple preprocessing components occasionally results in minor performance trade-offs. For example in Table 7 ensemble SMOTE+SOD has a marginally lower f1 score (97.78%) than SOD alone (97.98%). Likewise in Table 8 where we ensemble SMOTE with SOD coming after it has a marginally lower F1 score(96.62%) than SMOTE alone (96.88%). Furthermore, the addition of the FE component in Table 8 resulted in stagnant performance. Scientifically, these trade-offs are a direct consequence of the sequencing of these methods, specifically applying resampling techniques (SMOTE or ADASYN) prior to Supervised Outlier Detection (SOD). Distance-based oversampling algorithms generate synthetic data by interpolating between existing minority instances; when applied to an uncleaned dataset, they inevitably utilize existing noise and outliers as reference points, thereby propagating and amplifying the noise. Consequently, when SOD is subsequently applied, the statistical distribution (e.g., the interquartile range) has already been distorted by the artificially generated instances. This distortion blurs the decision boundaries, causing the SOD filter to either miss amplified noise or inadvertently discard valid edge cases. Likewise, the FE step may discard features that hold unique predictive value for specific subsets of students. These observations acknowledge that maximal preprocessing does not unilaterally guarantee improvement in every intermediate configuration Regardless of the balance strategy, the results indicate strong performance when applying both SOD and FE, which supports SOD-FE as a strong approach to enhance classification performance in complex and different datasets.

SOD-FE operates within the proposed approach to boost model performance when working with the imbalanced datasets often found in educational contexts. The modular design enables effortless integration with institutional analytics systems while supporting interpretability. However, this study contains limitations. Although the SOD-FE method offers various benefits, its analysis remains univariate because it depends on Pearson correlation to identify linear relationships. The analysis method has the potential to miss complicated non-linear patterns between features. The data preprocessing phase needs further investigation to develop multivariate outlier detection approaches and non-linear feature relevance measures for enhancing its robustness and accuracy.

## Discussion

The experimental evaluation demonstrates that the proposed SOD-FE method significantly enhances student dropout prediction across various machine learning models and datasets. When combined with the Random Forest classifier, the SOD-FE approach achieved high F1 scores on both datasets–98.09% for the Portugal dataset and 98.33%. Quantitatively, the SOD-FE framework demonstrated substantial superiority over existing approaches. On the Portugal dataset, it outperformed the highest-performing a CNN-Attention-XGB hybrid^38^ by 1.09% in F1-score. More strikingly, on the Slovakia dataset, the approach yielded a 7.33% improvement in F1-score over prior Random Forest implementations^26^, confirming the critical value of label-aware outlier filtering combined with targeted feature engineering. SOD played a crucial role in preserving informative outlier cases related to dropout by eliminating anomalies based on the relationships between features and the target variable. It can be noted that the high F1 scores are due to the curated nature of these datasets with the availability of academic features rich in information, coupled with the removal of noisy outliers from the datasets. Hold-out validation along with stratified 5-fold cross-validation was used to ensure that students were not repeated in both training and test sets to prevent potential overfitting. The generalizability was shown by obtaining similar results when using the SOD-FE framework on different datasets. These datasets vary in terms of student demographics, institutional structure and socio-economic context. High performance was obtained by the proposed method on the Portugal and Slovakia datasets. However, the dataset was small (261 students) which may hinder generalizability to all students. Adding the Portugal dataset helped to compensate for this. Future work should test the model on more extensive and more diverse datasets to make it robust and scalable. Besides, model interpretability tools like SHAP offered useful information about the predictors of dropouts. These insights support the practical deployment of predictive models to assist educational institutions in identifying and supporting at-risk student. While the IQR filter with Pearson correlation primarily captures linear relationships, our SOD-FE approach mitigates this by integrating Mutual Information, which effectively identifies non-linear dependencies. However, we acknowledge that multivariate anomaly detection techniques, such as Isolation Forests or Autoencoders, could further capture complex multidimensional outliers. Future work should explore the integration of non-linear feature analysis methods to enhance robustness and generalizability.

### Limitations

Despite the SOD-FE approach demonstrates significant predictive capabilities, its practical deployment in real-world educational environments faces certain implementation barriers. First, as a strictly supervised framework, SOD-FE heavily relies on the availability of high-quality, historically labeled data, which may not always be accessible in real-world educational environments. Incomplete or biased historical records can propagate inaccuracies throughout the filtering and modeling stages. Second, while the framework is highly efficient during the inference phase, allowing for rapid real-time predictions for new students. The initial training and periodic retraining phases demand considerable computational resources. Specifically, the iterative feature selection and exhaustive grid-search tuning must be completely re-executed whenever the institution updates the model with new semester data. This periodic retraining overhead may pose logistical challenges for institutions relying on standard computing hardware. Finally, the current iteration of the SOD method relies on univariate IQR-based filtering. While it effectively cleans noise feature-by-feature, this approach may inadvertently miss complex, multivariate anomalies. Future work could investigate multivariate anomaly detection baselines (i.e., Isolation Forests) and more scalable optimization methods to address these issues.

### Ethical considerations and practical deployment

Although the suggested model has a high predictive accuracy, its implementation in real-world education needs a close attention to both practical and ethical considerations. False positives can result in unwarranted interventions or unwanted stigmatization, whereas false negatives may result in missed opportunities to support at-risk students. Moreover, biases in the raw data can cause different treatment of groups of students especially in case sensitive attributes are implicitly encoded in the features. It is worth noting that the model must exclusively be used as a decision-support tool, but not as a decision-making system. The human intervention is necessary to make predictions readable and make sure interventions are used in an ethical and proper manner. Transparency is also achieved through the integration of explainable artificial intelligence methods, including SHAP, which allows one to understand how features contribute to the global and local level and hence make informed and accountable interventions in education.

## Conclusion

This study proposed a supervised outlier detection and feature engineering framework (SOD-FE) for recognizing student dropout prediction in higher education. The SOD-FE approach improved multiple machine learning (ML) models through its targeted methods for dealing with class imbalance problems and handling irrelevant or noisy data using outlier filtering and systematic feature engineering approaches. The combination of outlier filtering using the SOD and improved feature selection mechanisms based on RFI and RFE has created better performing and understandable models. The component-wise analysis further confirms the contribution of each stage in the proposed pipeline, while also revealing important trade-offs between noise reduction and information preservation. In addition, the integration of explainable artificial intelligence techniques (SHAP) provides both global and local interpretability, enabling a deeper understanding of model decisions and misclassification patterns. The experimental results on the Portugal and Slovakia benchmark datasets demonstrate that SOD-FE consistently improves classification performance, achieving F1 scores of up to 98.33%, and outperforming baseline models and recent approaches in the literature. The SOD-FE approach presents a robust and effective solution for early student dropout prediction. However, future academic research needs to collect data from multiple higher education institutions and apply advanced multivariable and non-linear analysis methods to improve prediction accuracy and the generalizability of the results. Moreover, our proposed study has developed an approach that incorporates ML and neural network-based techniques. Embedding with advanced models such as CNNs, LSTM networks, and vision transformers can also be researched. These models are useful in sequential dependency modeling patterns, which can be more robust, adaptable, and comprehensive for analyzing the risk of student dropout.

## Data Availability

The data presented in this study are publicly available at: https://archive.ics.uci.edu/dataset/697/predict+students+dropout+and+academic+success (accessed on 28 March 2026). Additional data are available at: https://github.com/JKabathova/Students-Dropout-Prediction (accessed on 28 March 2026). Code availability: The Python scripts and machine learning workflows used to process the datasets and implement the SOD-FE framework are publicly available on GitHub at: https://github.com/AlzabidiEissa/SOD-FE_Student_Dropout.git

## Code availability

The Python scripts and machine learning workflows used to process the educational datasets and implement the SOD-FE framework are available on GitHub at: https://github.com/AlzabidiEissa/SOD-FE_Student_Dropout.git.
